# Macroalgae *Sargassum cristaefolium* Extract Inhibits Proinflammatory Cytokine Expression in BALB/C Mice

**DOI:** 10.1155/2020/9769454

**Published:** 2020-10-09

**Authors:** Eka Sunarwidhi Prasedya, Ni Wayan Riyani Martyasari, Angga Susmana Abidin, Sonia Ardilla Pebriani, Bq Tri Khairina Ilhami, Andri Frediansyah, Anggit Listyacahyani Sunarwidhi, Sri Widyastuti, Haji Sunarpi

**Affiliations:** ^1^Bioscience and Biotechnology Research Centre, Faculty of Mathematics and Natural Sciences, University of Mataram, Mataram, Indonesia; ^2^Department of Pharmacy, Udayana University, Jimbaran, Bali, Indonesia; ^3^Pharmaceutical Institute, University of Tuebingen, Tuebingen, Germany; ^4^Research Division for Natural Product Technology (BPTBA), Indonesian Institute of Sciences (LIPI), Wonosari, Indonesia; ^5^Department of Pharmacy, Medical Faculty, University of Mataram, Mataram, Indonesia; ^6^Faculty of Food Technology and Agroindustry, University of Mataram, Mataram, Indonesia

## Abstract

Ultraviolet radiation (UVR) which could induce skin damage and skin disease is a growing concern due to the increase in global warming. Brown macroalgae *Sargassum cristaefolium* has been recognized to exhibit UV protective activities. However, the mechanism of its photoprotective activity remains unclear. The purpose of this study is to investigate the potential mechanism of *S. cristaefolium*'s photoprotective activity against UV radiation. Phytochemical analyses revealed valuable bioactive compounds in SCE, such as fucoxanthin which is widely known as an anti-inflammatory carotenoid. Treatment with SCE before UV-A radiation show reduced levels of wrinkles and desquamation. Interestingly, SCE treatment induces the skin healing process after UV radiation. SCE effectively inhibited proinflammatory TNF-*α* and IL-6 expression while increasing IL-10 production in the BALB/*c* mice skin. Current results suggest that SCE potentially protects the skin by attenuation of inflammatory cytokines. In addition, SCE demonstrates promising antibacterial activity (MIC = 1.302 *µ*g/mL) against *Staphylococcus aureus.* Overall, SCE could be a source of an effective anti-inflammatory agent protecting against UV irradiation-induced skin damages.

## 1. Introduction

Ultraviolet (UV) radiation is a major cause of skin aging, which is characterized by wrinkles, collagen formation, roughness, and loss of skin tone [1–3]. There are three types of UV rays, UV-A, UV-B, and UV-C, which are classified according to their wavelength. Of these, Ultraviolet-A (UV-A) penetrates deeper into the epidermis and dermis of the skin, which consist of keratinocytes and fibroblasts [4].

Ultraviolet-A induces skin aging in a variety of mechanisms, including oxidative stress, inflammation, and extracellular matrix degradation. For instance, the increased production of Reactive Oxygen Species (ROS) can lead to the cellular accumulation of damaged biomolecules, such as cellular proteins, lipids, RNA, and DNA [5, 6]. UV exposure of skin also promotes the production of inflammatory cytokines, such as tumor necrosis factor-*α* (TNF-*α*), interleukin (IL)-1*β*, and IL-6. These inflammatory cytokines would, furthermore, contribute to the accumulation of ROS which could lead to tissue and cell damage [7, 8]. Hence, the levels of proinflammatory cytokines may provide critical information on acute skin inflammation severity.

Macroalgae are one of the most extensively studied marine organisms. Previous studies have reported biological activities, including antioxidant, anticancer, antihypertension, hepatoprotective, immunomodulatory, and neuroprotective activities [9, 10]. Macroalgae are not only known to possess bioactive compounds associated with skincare against UV radiation, including polysaccharides, proteins, lipids (PUFA, sterols, and squalene), minerals, and vitamins, but also the secondary metabolites such as phenolic compounds, terpenoids, and halogenated compounds [11]. Extracts derived from *Sargassum* macroalgae reportedly exhibit antioxidant, antimicrobial, and UV protective properties [12]. Another research also described the potential of *Sargassum* macroalgae as a rich source of anti-inflammatory substances [13].

In our previous work, *Sargassum cristaefolium* ethanol (SCE) extract showed potential photoprotective activity against UV-A radiation [14]. However, the underlying mechanisms of SCE UV photoprotective activity have not been studied and remain largely unresolved. Therefore, the purpose of this study is to evaluate the effects of SCE against UV-A-induced proinflammatory responses *in vivo* in BALB/*c* mice and to further elucidate the possible mechanism for skin protection.

## 2. Materials and Methods

### 2.1. Sample Collection and Extraction

Brown macroalgae *Sargassum cristaefolium* were collected from the west coastal area of Lombok (8°29′54.251″ S and 116°4′36.664″ E). The macroalgae were identified regarding the algae electronic database [15]. After collection, the samples were directly rinsed with seawater to remove sand debris. Samples were, then, dried under room temperature conditions for 5 days without direct exposure to sunlight. Macroalgae dry samples were ground and dissolved in absolute ethanol solvent 5x volume of the sample weight (w/v). Mixed solutions were incubated at room temperature for the 48 h maceration process followed by stirring every 6 hours. After 24 hours, the mixed solutions were filtered with Whatman grade 1 filter papers. Finally, macroalgae extracts were subjected to the rotary evaporator (Sanjing, China) to evaporate the remaining ethanol solvents. Obtained filtrates were, then, used as macroalgae ethanol extracts for further experiments.

### 2.2. Chemical Analysis of Bioactive SCE

The identification of bioactive compounds was determined from *Sargassum cristaefolium* extract (SCE). The MS spectra of MS1 and MS2 were collected by using the maXis 4G of High-Resolution Time-of-Flight Mass Spectrometer (HR-TOF-MS) with electrospray ionization (Bruker Daltonic GmbH, Bremen, Germany). About 5 *µ*l of the nonpolar fraction was subjected to the reversed phase of the C18 column (Phenomenex Luna Omega Polar 5 *µ*m C18 100 Ă 4.6 × 150 mm) with a flow rate of 0.75 ml min to disperse the analytes. MS analysis was performed in the positive mode (50 eV). A grade from 90% water containing 0.1% formic acid to 100% acetonitrile with 0.06% formic acid for 40 min run was applied. The eluent was detected by ESI-MS monitoring m/z 100–1600. Peaks were analyzed using software Bruker Compass Data Analysis 4.4 SR1. The exact mass, together with the predicted molecular formula, was, then, subjected to Scifinder, PubChem, and METLIN (Scripps Research, La Jolla, California, USA).

To confirm the predicted compound, the MS2 that contains a fragmented pattern was, then, subjected to GNPS (Global Natural Product Social Molecular Networking) [16]. Firstly, the peaks from raw data were selected based on the intensity threshold of 100000 counts with a minimum peak length of spectra 2 to provide a consensus of spectra. Subsequently, it was converted to the format of.mzxml. The MS2 data were dereplicated using high-throughput comprehensive MS/MS libraries from GNPS in combination with metadata of spectrum (MASST). The specific search options were selected using a precursor ion mass tolerance of 0.5 Da, fragment ion tolerance of 0.5 Da, minimal match six, and score threshold of 0.7. The search analog function was also turned on. The rest of the options were set to default. The HRMS data of SCE is publicity available and have been deposited in the Center for Computational Mass Spectrometry, University of California, San Diego, USA, with ID MSV000085323 (doi: 10.25345/C5GH69).

### 2.3. Antibacterial Activity SCE

The antibacterial activity analysis of the crude extract from *S. cristaefolium* against bacterial skin infection from *Staphylococcus aureus* was performed in a 96-well plate using the broth dilution method according to The European Committee on Antimicrobial Testing (EUCAST) guideline [17]. Briefly, 190 *µ*l of the Mueller-Hinton broth and 10 *µ*l of each bacterial pathogen with OD600 = 0.2 were cultured in each well. A total of 200 *µ*L from the solution of 128 *µ*g/mL of crude extract in DMSO was applied in the first vertical line of the 96-well plate. Serial dilutions were constructed to obtain twelve different concentrations in each well starting from 64 *µ*g/mL. DMSO without any additional chemical was used as the negative control. The 96-well plates were, then, incubated for 24 h at 37°C, and growth inhibition was measured using a Tecan Plate reader Infinite M200 at 600 nm. The MIC was determined in independent triplicate for each bacterial pathogen. The OD600 data that represented bacterial growth were adjusted by the Gompertz equation to calculate the MIC [18].

### 2.4. Animals

For this experiment, a total of 30 female BALBL/*c* mice, 6–8-week-old and weighing 25–30 g, were used. The mice had free access to food and water in a temperature-controlled (24–25°C) and humidity-controlled (50%) room. Animals were also subjected to a 12 h light/dark cycle. All experimental procedures complied with the Health guidelines for Care and Use of Laboratory Animals and were approved on 15 December 2019 by the Bioethics Committee of Medical Faculty University of Mataram.

### 2.5. *In Vivo* UV-A Irradiation

Mice were randomly divided into 3 groups (*n* = 6 per group) that represented treatments and controls. These were normal healthy controls with no UV treatment (NC), normal healthy controls with UV treatment (VC), and UV irradiation with 0.1% SC treatment (SCE). The choice of concentration for animal experiments is based on the previous work which shows that 0.1% concentration of SCE shows significant protective activities against UV radiation [14].

Before experimentation, mice were anesthetized by intraperitoneal injection of 1% sodium pentobarbital, and hair was removed from the back skin of mice using hair removal wax. Parallel UV lamps Reptile UV150 PT2188 13W (Exo Terra, Winchester, MA, USA) were used for UV-A irradiation treatment. The distance from the UV lamp to mice was 20 cm to obtain a UV-A spectral radiance of 300 *μ*W/cm^2^. Animals were irradiated for 7 days, 2 h/day, with a daily dose of 2.16 J/cm^2^. SCE was treated to exposed skin (2 cm^2^) after UV-A exposure to evaluate SCE activity in reducing inflammation responses.

### 2.6. Images Records of UV-A-Irradiated Dorsal Skins

Experimenters took photos of every treated mouse dorsal skin area every day for the entire 7-trial treatment. Image records were taken 1 hour after UVR-treated mice were administered with SCE. The macroscopic visual scores of wrinkles were calculated by blinded investigators according to the grading scale by Wang with slight modifications, as shown in Table 1 [9]. Erythema and desquamation (scaling) scoring from 1–4 were conducted based on the Psoriasis Area Severity Index (PASI) [19].

### 2.7. Hematoxylin and Eosin (H&E) Staining

Skin specimens were fixed in 10% formaldehyde and embedded in paraffin. The sections were, then, deparaffinized in xylene, rehydrated through a graded alcohol series, and stained with H&E (Biosharp, Wuhan, China) according to the manufacturer's protocol. Pathological changes were observed under an inverted microscope (Zeiss, Gottingen, Germany).

### 2.8. Immunohistochemistry (IHC)

The mice skins were isolated, fixed in 4% paraformaldehyde (PFA) overnight at 4°C, and embedded in paraffin (26). The brain sections were, then, deparaffinized in xylene, rehydrated through a graded ethanol series, and subjected to antigen retrieval by boiling the slices in citrate buffer (pH 6.0) with high heat for 15 min and medium heat for 15 min in a microwave oven. For the IHC analysis, the sections were treated with 3% H_2_O_2_ for 10 min to remove endogenous peroxidase, blocked with 1% bovine serum albumin (BSA) in PBS (blocking solution) at room temperature for 1 h, and incubated with anti-TNF-*α* antibodies (Fine Biotech, Wuhan, China) diluted (1 : 200) in a blocking solution at 4°C overnight. After washing 3 times in PBS, the sections were incubated with HRP-conjugated secondary antibody (DAKO, Glostrup, Denmark) at room temperature for 30 min and, then, stained with 3ʹ-diaminobenzidine (DAB) for 15 s. Hematoxylin was used for cell nuclei detection. The stained sections were visualized and digitally scanned with an inverted light microscope (Zeiss, Germany).

### 2.9. Semiquantitative PCR Analysis

The expressions of proinflammatory cytokines TNF-*α*, IL-6, and anti-inflammatory cytokine IL-10 were examined using primers corresponding data in GenBank. The primers were ordered from Fasmac, Japan. Primer specifications are available in Supplementary Materials, Table S1. Total RNA was extracted from the dorsal skin tissues using the Qiagen RNeasy mini kit (Qiagen, USA). The cDNA was synthesized from equal amounts of total isolated RNA with the PrimeScript 1st strand cDNA synthesis kit (Takara, Japan), and PCR was performed using a Toptaq master mix PCR kit (Qiagen, USA). The quantities of PCR product relative to the housekeeping gene (GAPDH) were calculated with ImageLab software (Biorad, USA).

### 2.10. Statistical Analyses

Statistical analyses were conducted using KaleidaGraph 4.5.4 (Synergy Software, Reading, PA, USA) with two-tailed unpaired Student's t-test and one-way analysis of variance (ANOVA) followed by the Tukey–Kramer test. The data are presented as means ± standard deviation (SD). Differences among comparisons were considered statistically significant for *P* values less than 0.05.

## 3. Results

### 3.1. Macroalgae *Sargassum cristaefolium* Collection and Chemical Analyses

**Figure 1:**
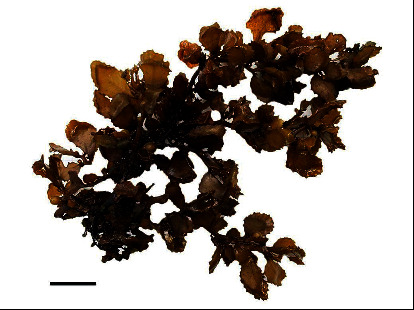
Morphological features of *Sargassum cristaefolium*.

Macroalgae specimens were collected in the mid of 2019 (Figure 1) and identified according to electronic algae database [15]. The goal of the bioactive analysis using MS1 and MS2 spectral data is to determine the spectral data of the bioactive target against spectral data of known substance in the database and literature (Table 2). Dereplication without MS1 will lead to unreliability and ambiguity due to the isobaric molecules. Fragmentation based on MS2 is more powerful to support the result from MS1 analysis. The dominant compounds are fucoxanthin and fatty acids such as 2-monoolein and 13-docosenamide. Fucoxanthin has been previously detected in various *Sargassum* species such as *Sargassum fulvellum*, *Sargassum heterophyllum, Sargassum horneri,* and *Sargassum siliquastrum* [20]. However, this is the first report to show its presence in *Sargassum cristaefolium* by LC-MS analysis.

### 3.2. Antibacterial Activity of SCE

**Figure 2:**
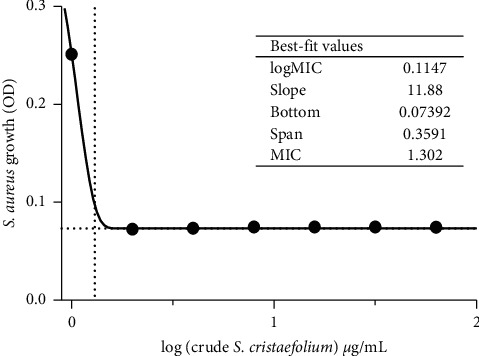
MIC graph of *S. cristaefolium* against extract *S. aureus* using the Gompertz equation. The antibacterial activity of *S cristaefolium* extract was determined by the broth microdilution method, with three independent replications. The MIC of its crude extract against *S. aureus* was 1.302 *µ*g/ml. DMSO as a control negative has MIC >500 *µ*g/ml.

The SCE extract was tested for its antimicrobial activity against *Staphylococcus aureus* to evaluate its possible clinical application (Figure 2). The antibacterial activity of SCE was determined by the broth microdilution method, with three independent replications. The MIC of its crude extract against *S. aureus* was 1.302 *µ*g/mL. DMSO as a control negative, has MIC >500 *µ*g/mL. Fucoxanthin is known as a carotenoid produced by brown macroalgae and is abundant in SCE. However, evaluation of SCE effects against other bacteria should be considered for further understanding of its antibacterial activity.

### 3.3. Pathological Changes in the UV-A-Irradiated Skin Surface

**Figure 3:**
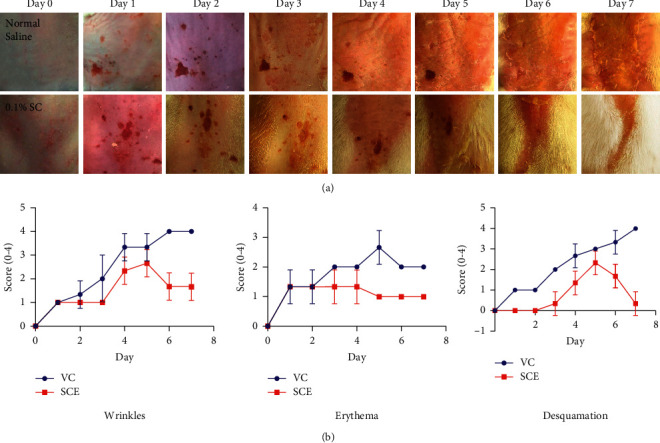
Pathological changes of the mice dorsal skin area after 2 h/day UV-A irradiation for 1 week. (a) The photo observations of the dorsal skins. Image records were taken of the dorsal skin area every day after 1 hour UV-A irradiation. (b) The graph showing wrinkles scoring. (c) The graph showing erythema scoring. (d) The graph showing desquamation scoring. Erythema and desquamation (scaling) scoring from 1–4 were conducted based on the Psoriasis Area Severity Index (PASI).

During the 7-day trial, the severity of the skin lesions conditions was scored every day using the erythema and scaling parts of the Psoriasis Area Severity Index (PASI). Wrinkles were scored based on the grading scale described in the study conducted by Wang with slight modifications. The representative photos of UV-A-induced mice dorsal skin areas are shown in Figure 3(a). During the 7 day treatments, wrinkles, erythema, and desquamation appeared frequently in these two groups. However, the severity of the VC group was higher in all categories compared to the SCE group. Although both groups developed wrinkles on day 4, the wrinkles in the SCE group were decreased after day 6 (Figure 3(b)). The anti-inflammatory properties of SCE also potentially contributed to the low erythema severity in the SCE group (Figure 3(c)). Another interesting feature was that, during the 6th day, SCE treatment showed an improvement in the wound and healing rate of skin lesions (Figure 3(d)).

### 3.4. SCE Prevents UV-A-Induced Epidermal Thickening

**Figure 4:**
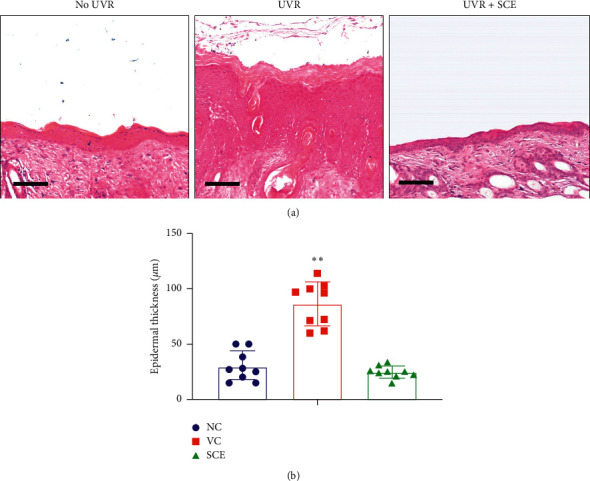
Evaluation of epidermal hyperplasia of the mice dorsal skin due to UV-A irradiation. (a) Hematoxylin and eosin (H&E) staining of UV-A-irradiated mice dorsal skin. (b) Epidermal thickness of the mice skin dorsal area, analyzed with Zeiss Axiovision software. Scale: 50 um. ^*∗∗*^Highly significantly different compared to control (NC); *P* < 0.01.

The histological features of the dorsal skin were analyzed with hematoxylin and eosin (H&E) staining at 7 days after UV-A exposure (Figure 4). Exposure to UV-A radiation causes skin symptoms such as skin inflammation, including erythema, edema, and epidermis thickening. Epidermal thickening can be used to reflect photoaged skin since it can result in skin roughness and wrinkles. As illustrated in Figure 4(a), there is a remarkable difference of epidermal thickness found between groups not treated with UV (NC) and the group irradiated with UV with no extract treatment (VC). Epidermal thickening was markedly decreased to 20 *µ*M in SCE treated groups after UV radiation (Figure 4(b)).

### 3.5. SCE Inhibits Expression of UV-A Induced Proinflammatory Cytokines

**Figure 5:**
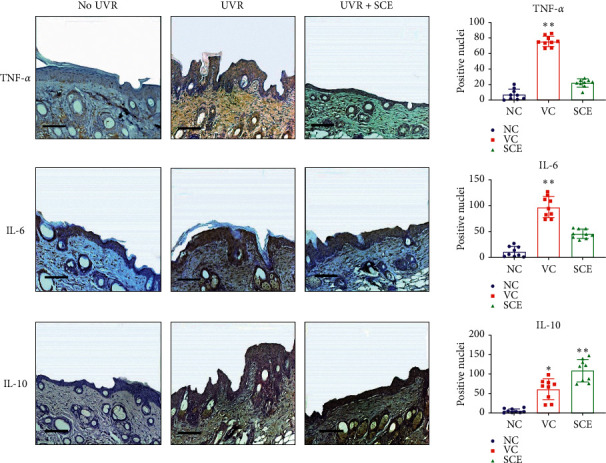
Immunohistochemistry analyses of target proteins TNF-*α*, IL-6, and IL-10. Scale: 50 um. ^*∗∗*^Highly significantly different compared to control (NC); *P* < 0.01.

The effect of SCE treatment on UV-A-induced damaged inflammatory cytokines of mice dorsal skins was evaluated with immunohistochemistry (IHC) and semiquantitative PCR. In the UVR-treated group (VC), inflammatory cytokines TNF-*α* and IL-6 immunoreactivity were dramatically increased compared to the NC negative control group (Figure 5). However, SCE shows inhibitory effects on proinflammatory production. This was shown by the number of TNF-*α* positive nuclei that were significantly reduced after treatment with SCE. On the contrary, anti-inflammatory cytokine IL-10 production was increased after SCE treatment. The production of IL-10 is delayed and always follows that of proinflammatory factors with a latency of a few hours.

**Figure 6:**
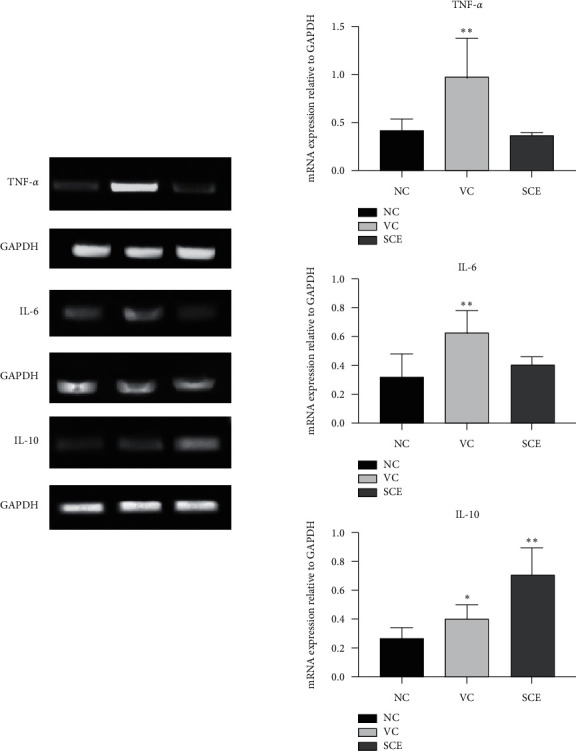
Semiquantitative PCR analyses for determination of mRNA level expression of target genes TNF-*α*, IL-6, and IL-10. ^*∗∗*^Significantly different compared to control (NC); *P* < 0.05. ^*∗∗*^Highly significantly different compared to control (NC); *P* < 0.01.

The effect of SCE on mRNA expressions of proinflammatory cytokines TNF-*α* and IL-6 was analyzed using semiquantitative reverse transcriptase PCR (Figure 6). In correlation to IHC results, mRNA expressions of TNF-*α* and IL-6 significantly decreased in SCE-treated groups compared to control groups (NC). The anti-inflammatory IL-10 mRNA levels were increased also in SCE groups. These results indicate that SCE potentially increases IL-10 production which regulates the overexpression of proinflammatory cytokines TNF-*α* and IL-6 due to UVR exposure.

## 4. Discussion

Marine macroalgae are efficient primary producers and an important source of natural bioactive products, especially macroalgae from the intertidal zone which have to withstand multiple changes in abiotic factors due to tidal changes [21]. Brown macroalgae *Sargassum* is one example of such macroalgae in the intertidal zone which has to cope with temporarily high ultraviolet radiation (UVR). Numerous studies have demonstrated various pharmacological properties of the *Sargassum* species [22, 23]. However, information regarding this group of species' biological activity is very limited. In the present study, we investigated the effects of an extract derived from *Sargassum cristaefolium* (SCE) on UVR-induced inflammatory cytokines.

Phytochemical analysis revealed an abundance of fucoxanthin in SCE. Fucoxanthin is a major carotenoid found in most edible brown seaweeds, such as *Undaria pinnatifida*, *Hijikia fusiformis*, and other *Sargassum* species such as *Sargassum fulvellum* [20]. However, this is the first report to show fucoxanthin's presence in *Sargassum cristaefolium* ethanol extract. Recently, there have been several reports that show beneficial health effects of fucoxanthin such as cancer prevention, antidiabetes, and antiobesity [24]. Fucoxanthin also has antioxidative, anti-inflammatory, and antiangiogenic effects which potentially contribute to its UV protective activity [25]. Also, a recent study shows UVR photoprotective activity of fucoxanthin extracted from brown algae *Undaria pinnatifida* [26]. Furthermore, fucoxanthin is also known as an antibacterial carotenoid [27]. Hence, the presence of fucoxanthin in SCE potentially contributes to its antibacterial activity against *Staphylococcus aureus*. Antibacterial agents are widely applied in UV protection products to help the skin to stay clean and healthy [28].

Skin aging is often attributed to various processes such as increased wrinkles, erythema, desquamation, loss of tensions and elasticity, altered pigmentation, and so forth [29]. In this study, we found that SCE could inhibit wrinkle formation, erythema, and desquamation due to UVR exposure. Chinese people use various species of *Sargassum* to treat scrofula, edema, arteriosclerosis, and skin diseases [23]. Previous studies also demonstrated utilizing *Sargassum* as an anti-inflammatory agent associated with its application for acute and chronic inflammation [13]. For example, *Sargassum binderi* shown elevated wound and healing rates in streptozotocin-induced diabetic rats [30]. Another study also presented that *Sargassum muticum* ethyl acetate fraction could inhibit wrinkle formation in mice [31].

Earlier findings have confirmed that repeated UV exposures of the skin would lead to epidermal hyperplasia [32]. Epidermal thickening is an inflammatory response caused by hyperproliferation and aberrant differentiation of keratinocytes or leukocytes which infiltrate into the dermis and consequent cytokines and chemokines production [33]. The suppression of epidermal thickening by SCE treatment shows its potential anti-inflammatory activity. The previous report has presented anti-inflammatory effects of *Sargassum polycystum* on lipopolysaccharide- (LPS-) stimulated RAW 264.7 macrophages [13]. The UV response involves activation of numerous signal transduction pathways which play critical roles in the regulation of cell division and death. Another study reported that *Sargassum fusiforme* could act as an inducer of the Nrf2-ARE pathway which mainly regulates anti-inflammatory gene expression and inhibits the progression of inflammation [34]. Further studies regarding SCE effects on these pathways would be interesting to understand more about its photoprotective mechanism.

UV radiation leads to inflammatory reactions such as epidermal cell cytokine production as an immune response. Much of the UV response is believed to be due to the activation of MAPK and NF*κ*B pathways [35]. The transcription factor NF*κ*B is a key regulator of TNF production and TNF-induced proinflammatory gene expression. TNF-*α* is a proinflammatory cytokine that controls multiple cellular processes, such as the production of downstream inflammatory mediators such as IL-3, IL-1*β*, and IL-6 [36]. These inflammatory factors mediate the growth of epidermis which leads to epidermal hyperplasia. Hence, inhibition of these proinflammatory cytokines by SCE treatment resulted in reduced epidermal thickness. Besides, SCE treatment potentially increases IL-10 expression. It is notable that IL-10 downregulates proinflammatory cytokines such as TNF-*α*, IL-6, and IL-1 [37]. A previous study has reported that IL-10 enhances healing in wounds which possibly explains the healing activity of SCE [38]. *Sargassum siliquastrum* revealed anti-inflammatory activity in the LPS-induced 264.6 RAW cells via potential downregulating the transcription of proinflammatory cyclooxygenase-2, prostaglandin E2, and TNF-*α* via MAPK pathway inhibition [39]. Another *Sargassum* species, *Sargassum horneri*, revealed the presence of sulfate esters and fucose which were involved in anti-inflammatory potential via decreasing the TNF-*α* secretion and NO production [40].

## 5. Conclusions

In conclusion, the present study demonstrates the anti-inflammatory activity of *Sargassum cristaefolium* ethanol extract (SCE) by potentially attenuating the inflammatory cytokines. SCE showed the presence of fucoxanthin with antibacterial and anti-inflammatory activities. Histopathological analyses also showed that SCE treatment reduced epidermal thickness due to UVR induced skin inflammation. SCE also demonstrated inhibition of proinflammatory cytokines TNF-*α* and IL-6 in skin lesions of UVR treated mice. Also, anti-inflammatory cytokine IL-10 levels were higher in mice treated with SCE after UVR which possibly contributes to faster regenerative healing properties. Current results show that SCE is a potential antiphotoaging agent for skin protection products. However, further investigations of purified compound level photoprotective activity should be considered in the future.

## Figures and Tables

**Table 1:** Grading scales for wrinkle scoring of the UV-A-treated mice skin.

Grade	Evaluation criteria
0	No wrinkles
1	Fine striations
2	Shallow wrinkles; the disappearance of all fine striations
3	Deep wrinkles across the dorsal skin
4	Severe wrinkles; development of lesions

**Table 2:** Summary of bioactive compounds analysis based on MS^1^ and MS^2^. The exact mass of HRMS spectra was compared with the available database of METLIN, Scifinder, PubChem, and GNPS. The MS2 data were submitted to high-throughput dereplication comprehensive MS/MS libraries from GNPS (Supplementary Material, Figure S1–S6).

(*M*+*H*)^+^ precursor ions (*m*/*z*)	Predicted molecular formula from (*M*+*H*)^+^/error (ppm)/ring double bond	Match with MS^1^ database	Match with MS^2^ database	Annotation
659.4306	C_42_H_59_O_6_/−0.1/14	Yes	Yes	Fucoxanthin
273.3001	C_21_H_41_O_4_/−0.4/2	Yes	Yes	2-Monoolein
338.3417	C_22_H_44_NO/0.2/2	Yes	Yes	13-Docosenamide
500.3951	C_28_H_54_NO_6_/−0.7/3	No	No	Unidentified 1
457.3999	C_26_H_53_N_2_O_4_/0.4/2	No	No	Unidentified 2
498.3795	C_28_H_52_NO_6_/−1.2/4	No	No	Unidentified 3

## Data Availability

The data sets generated and/or analyzed during the current study are available from the first author on reasonable request.

## Supplementary Materials

Supplementary Materials All of the additional experimental data, including the extracted chromatogram, structure of the detected bioactive compounds (Figures S1–S6), and specifications of primers used (Table S1), can be found in the supplementary materials.
