# Supplementary material for: Macroalgae Sargassum cristaefolium Extract Inhibits Proinflammatory Cytokine Expression in BALB/C Mice
Source: Scientifica (Cairo). 2020 Oct 9;2020:9769454. doi: 10.1155/2020/9769454 (PMC7569464; doi:10.1155/2020/9769454)
Supplement: Supplementary Materials — All of the additional experimental data, including the extracted chromatogram, structure of the detected bioactive compounds (Figures S1–S6), and specifications of primers used (Table S1), can be found in the supplementary materials. [file 9769454.f1.pdf]

Supplementary data

Figure S1. MS^1, MS^2 and structure of fucoxanthin in *S. cristaefolium*
Figure S2. MS^1, MS^2 and structure of 2-monoolein in *S. cristaefolium*
Figure S3. MS^1, MS^2 and structure of 13-docosenamide in *S. cristaefolium*
Figure S4. Extracted chromatogram, MS^1 and MS^2 of Unidentified 1
Figure S5. Extracted chromatogram, MS^1 and MS^2 of Unidentified 2
Figure S6. Extracted chromatogram, MS^1 and MS^2 of Unidentified 3
Table S1. Primer specifications

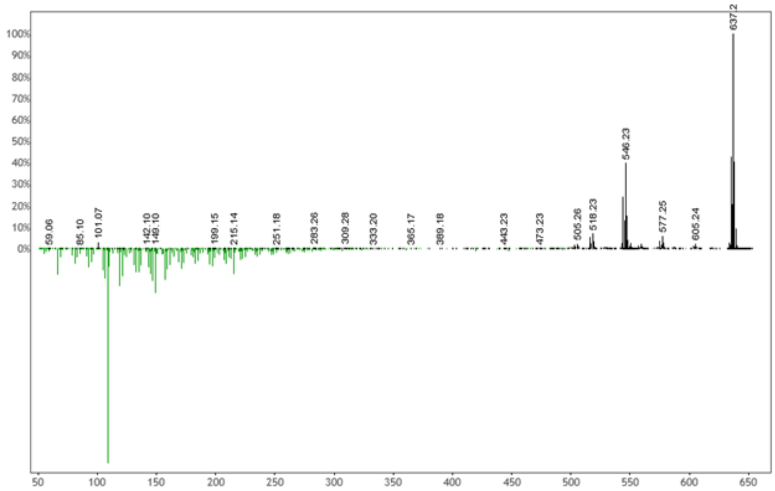

(a)

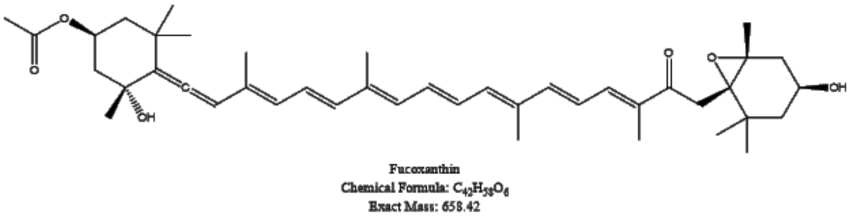

(b)

Figure S1. Identification of fucoxanthin from *Sargassum cristaefolium* with m/z 659.4306. (a) Mirror match MS^2 data with MASST spectrum from GNPS library (green line) with cosine score of 0.84. (b) fucoxanthin structure, formula, and exact mass.

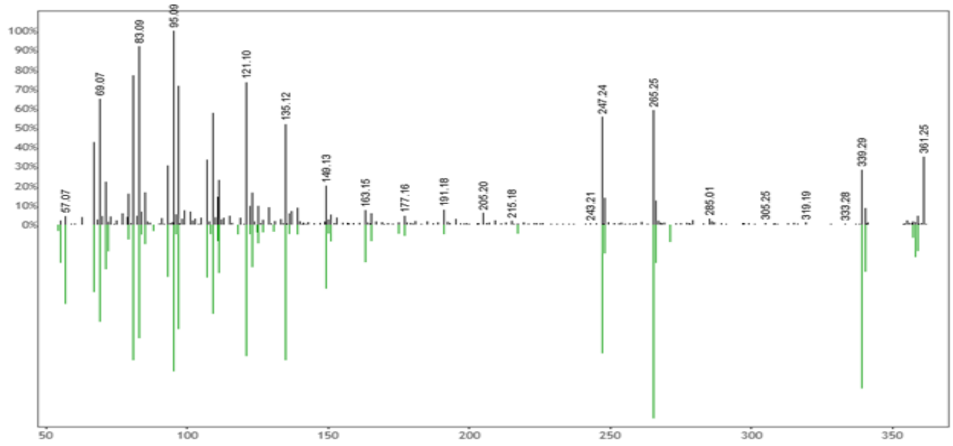

(a)

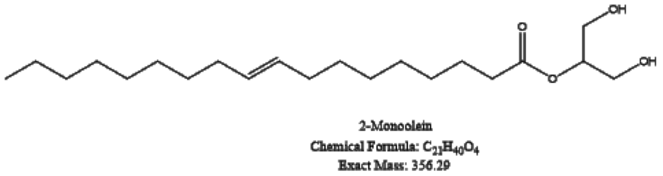

(b)

Figure S2. Identification of 2-monoolein from *Sargassum cristaefolium* with m/z 357.3001 (a) Mirror match MS^2 data with MASST spectrum from GNPS library (green line) with cosine score of 0.93. (b) 2-monoolein structure, formula, and exact mass.

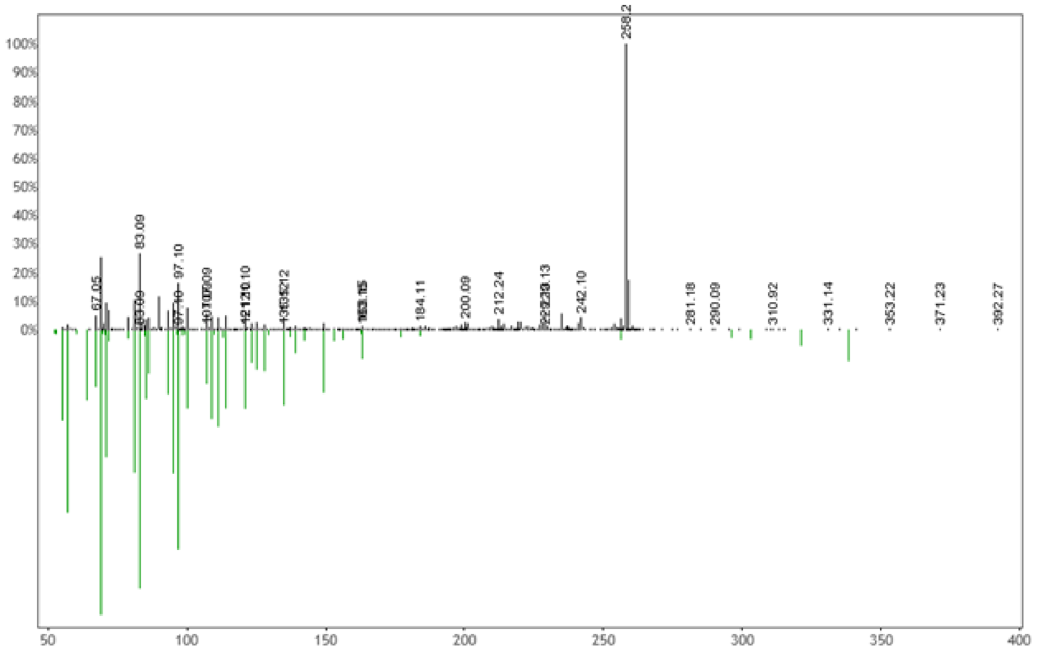

(a)

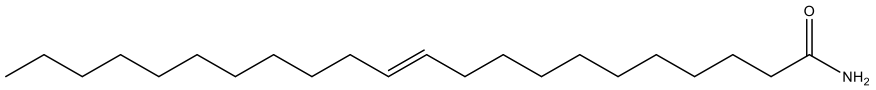

13-docosonamide
Chemical Formula: $C_{22}H_{43}NO$
Exact Mass: 337.33

(b)

Figure S3. Identification of 13-docosenamide from *Sargassum cristaefolium* with m/z 338.3417. (a) Mirror match MS^2 data with MASST spectrum from GNPS library (green line) with cosine score of 0.79. (b) 13-docosenamide structure, formula, and exact mass.

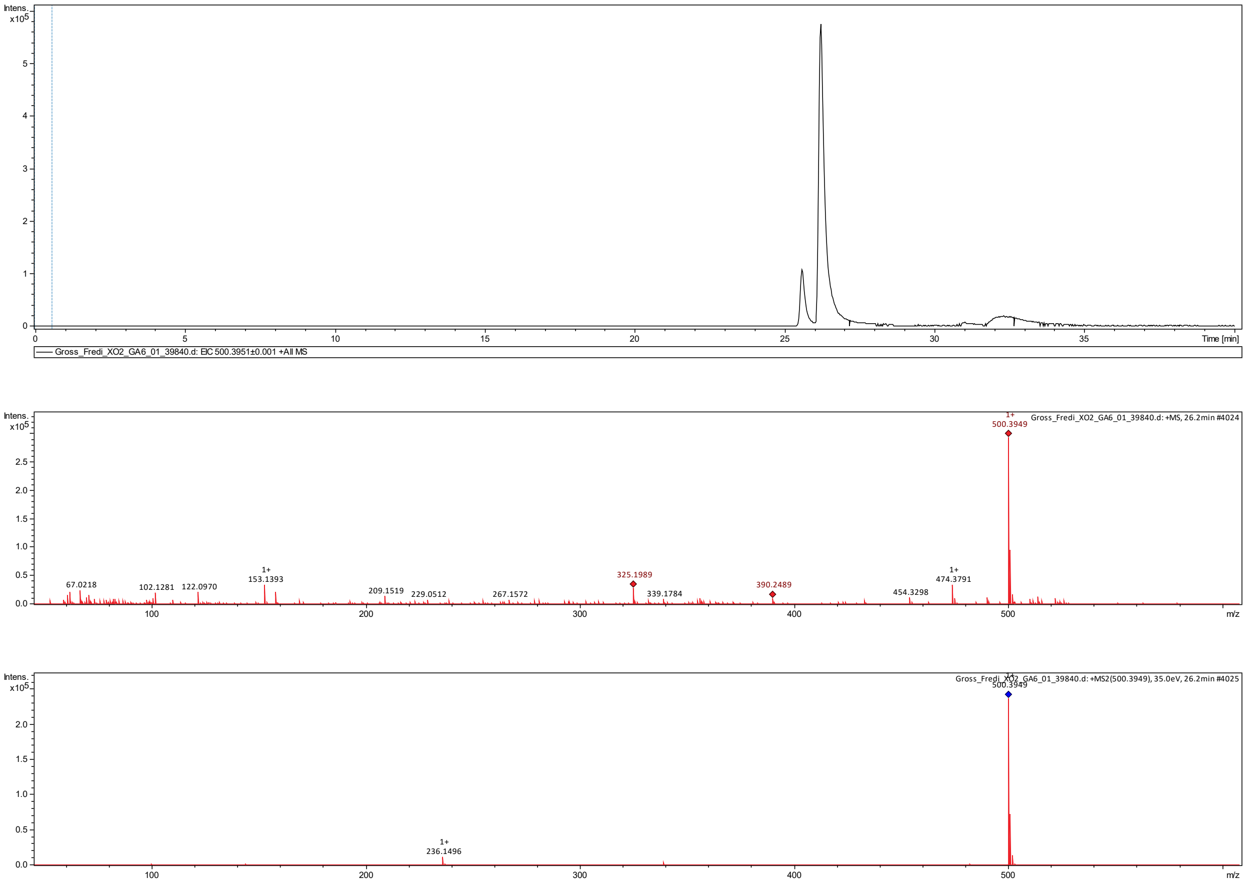

Figure S4. From up to down extracted chromatogram, MS^1 and MS^2 , respectively, of unknown compound with m/z 500.3951

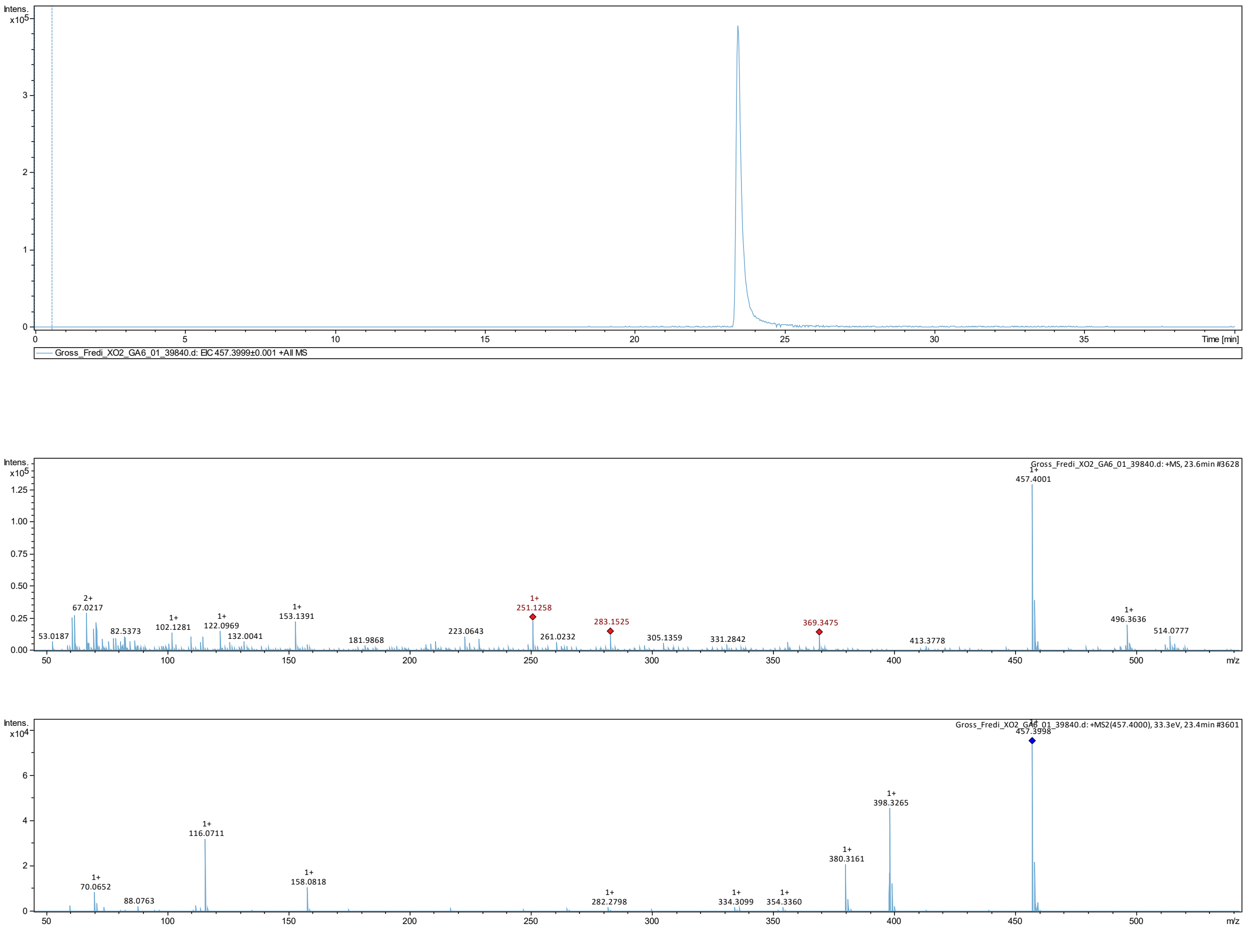

Figure S5. From up to down extracted chromatogram, MS1 and MS2, respectively, of unknown compound with m/z 457.3998

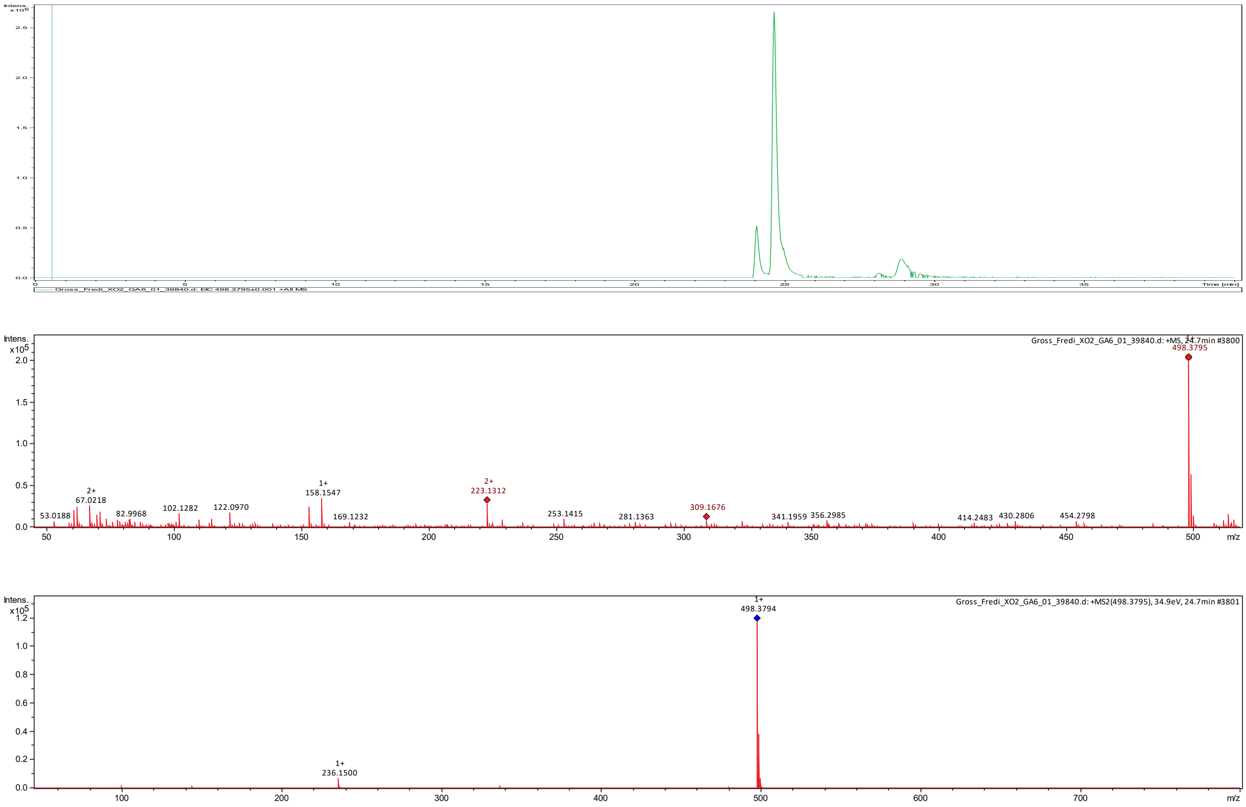

Figure S6. From up to down extracted chromatogram, MS1 and MS2, respectively, of unknown compound with m/z 498.3794.

Table S1. Primer Specifications

Primer	Sequence	Product size
TNF-α F	AGCCCCCAGTCTGTATCCTT	212 bp
TNF-α R	CTCCCTTTGCAGAACTCAGG	
IL-6 F	GAGCCCACCAAGAACGATAG	229 bp
IL-6 R	TCCACGATTTCCCAGAGAAC	
IL-10 F	CCAGGGAGATCCTTTGATGA	173 bp
IL-10 R	CATTCCCAGAGGAATTGCAT	
Gapdh	AACTTTGGCATTGTGGAAGG	223 bp
Gapdh	ACACATTGGGGGTAGGAACA	
